# Profiling small RNAs in fecal immunochemical tests: is it possible?

**DOI:** 10.1186/s12943-023-01869-w

**Published:** 2023-10-03

**Authors:** Einar Birkeland, Giulio Ferrero, Barbara Pardini, Sinan U. Umu, Sonia Tarallo, Sara Bulfamante, Geir Hoff, Carlo Senore, Trine B Rounge, Alessio Naccarati

**Affiliations:** 1 Centre for Bioinformatics, Department of Informatics, University of Oslo, Oslo, Norway; 2 Department of Clinical and Biological Sciences, University of Turin, Turin, Italy; 3 Department of Computer Science, University of Turin, Turin, Italy; 4 Italian Institute for Genomic Medicine (IIGM), c/o IRCCS Candiolo, Turin, Italy; 5 Candiolo Cancer Institute, FPO-IRCCS, Candiolo, Turin Italy; 6 Department of Pathology, Institute of Clinical Medicine, University of Oslo, Oslo, Norway; 7 Epidemiology and Screening Unit-CPO, University Hospital Città della Salute e della Scienza, Turin, Italy; 8 Section for colorectal cancer screening, Cancer Registry of Norway, Oslo University Hospital, Oslo, Norway; 9 Department of Research, Telemark Hospital, Skien, Norway; 10 Department of Research, Cancer Registry of Norway, Oslo, Norway; 11 Centre for Bioinformatics, Department of Pharmacy, University of Oslo, Oslo, Norway

**Keywords:** Small RNA sequencing, Fecal immunochemical test (FIT), Colorectal cancer screening, Stool biomarkers, Microbiome, microRNAs

## Abstract

Fecal microRNAs represent promising molecules with potential clinical interest as non-invasive diagnostic and prognostic biomarkers. Colorectal cancer (CRC) screening based on the fecal immunochemical test (FIT) is an effective tool for prevention of cancer development. However, due to the poor sensitivity of FIT especially for premalignant lesions, there is a need for implementation of complementary tests. Improving the identification of individuals who would benefit from further investigation with colonoscopy using molecular analysis, such as miRNA profiling of FIT samples, would be ideal due to their widespread use. In the present study, we assessed the feasibility of applying small RNA sequencing to measure human miRNAs in FIT leftover buffer in samples from two European screening populations. We showed robust detection of miRNAs with profiles similar to those obtained from specimens sampled using the established protocol of RNA stabilizing buffers, or in long-term archived samples. Detected miRNAs exhibited differential abundances for CRC, advanced adenoma, and control samples that were consistent for FIT and RNA-stabilizing buffers. Interestingly, the sequencing data also allowed for concomitant evaluation of small RNA-based microbial profiles. We demonstrated that it is possible to explore the human miRNome in FIT leftover samples across populations and envision that the analysis of small RNA biomarkers can complement the FIT in large scale screening settings.

**Supplementary Information:**

The online version contains supplementary material available at 10.1186/s12943-023-01869-w.

## Introduction

Colorectal cancer (CRC) is the third most common malignancy and the second leading cause of cancer-related deaths [1]. The promotion of healthy lifestyles and dietary choices, the development of new strategies for disease management, and the implementation of global screening programs are some of the strategies to reduce CRC morbidity and mortality.

Screening of selected age groups at risk is considered the most effective tool to prevent CRC development by detecting early tumor forms and precancerous lesions [2]. In many European countries, the first step of CRC screening relies on non-invasive stool-based tests such as the fecal immunochemical test (FIT). If the test is positive, patients are invited to visual examinations based on invasive endoscopic methods, such as colonoscopy. The advantage of first-line FIT is the relatively low-cost and ease of execution compared to colonoscopy. The FIT does not require specific preparation or dietary restriction and consequently has high acceptance rates. However, due to its poor sensitivity for premalignant lesions and the burden associated with an excessive number of colonoscopy procedures, different countries adopt thresholds for FIT positivity that are suited to their colonoscopy capacity, in a balancing act between sensitivity and specificity.

This highlights the need for alternative biomarkers to improve CRC screening accuracy. The implementation of complementary tests based on the analysis of the leftover of FIT stool samples could help improve in the identification of those individuals that would benefit from further investigation by colonoscopy. Both observational and experimental evidence point to a role for the gut microbiome in development and progression of CRC [3]. We have shown that it is possible to profile the microbiome in FIT leftover samples and archived stool samples [4]. However, larger discovery studies are needed to identify clinically valuable biomarkers [5]. Small noncoding RNAs (sncRNAs), particularly microRNAs (miRNAs), are detectable and stable in stool samples and are emerging as a candidate source of biomarkers for the non-invasive diagnosis of gastrointestinal diseases, including CRC [6]. Using small RNA sequencing, we have demonstrated the possibility to quantify the levels of both human and microbial sncRNAs in human stool samples. Interestingly, the combined use of human and microbial sncRNA levels was more efficient than using the two biomarkers alone in classifying CRC patients from colonoscopy-negative control subjects [7].

While we have shown that gut-derived miRNAs are potential biomarkers for CRC, little is known about the possibility of measuring them in a screening population by using the FIT buffer leftovers [8]. In this study, carried out in two independent European laboratories, we showed not only the feasibility of small RNA sequencing in FIT samples but we also tested the profiling robustness by comparing sequencing data in FIT samples with feces collected in stabilising buffers and long term archived fecal samples. In addition, we showed that some gut miRNAs differed in abundance between CRC/advanced adenomas (AA) and controls, suggesting a potential for discovering CRC biomarkers.

## Materials and methods

### Cohorts and samples

#### BCSN—FIT

Bowel Cancer Screening in Norway (BCSN) is an ongoing (2012–2023) randomized trial comparing once-only sigmoidoscopy with repeated FIT tests every second year for up to four rounds. The study is a pilot for the national screening program [9]. Stool samples are collected on plastic sticks designed to catch about 10 mg of stool and then stored in a 2ml buffer (Eiken Chemicals Ltd., Tokyo, Japan). Thirteen FIT samples from anonymized subjects participating in screening in 2015 were randomly selected for the purpose of this feasibility study. Collected samples were stored in their original tubes at -40˚C for up to 1 year.

#### NORCCAP—stool

The Norwegian Colorectal Cancer Prevention (NORCCAP) screening trial was carried out from 1999 to 2001 [10]. Participants were asked to bring a fresh frozen stool specimen collected at home less than one week before sigmoidoscopy and to keep it in a 20 ml vial in their home deep freezer (-20˚C) until attendance for flexible sigmoidoscopy. Eleven anonymized stool samples were randomly selected. Collected samples were stored without any stabilising buffer at -30˚C for approximately 17 years prior to RNA extraction.

#### MITOS—FIT and stool

The Italian biological samples have been collected in the frame of the regular Piedmont Region CRC screening in the Microbiome and MiRNA in Torino Screening (MITOS) project. The Piedmont Region screening program invites all residents, aged 59–69 to undergo a single sample biennial FIT (Eiken Chemicals Ltd., Tokyo, Japan). The collection of FIT leftovers for this study started in April 2017 and is still ongoing. A total of 185 subjects (classified based on colonoscopy results in 22 CRC, 80 AA, 30 non-advanced adenoma (nAA), and 53 controls) were included in the present study. Among them, 57 subjects (4 CRC, 25 AA, 6 nAA, and 22 controls) also provided stool samples before undergoing colonoscopy. In this case, stool samples were collected at home in nucleic acid collection and transport tubes with RNA stabilising solution (Norgen Biotek Corp.). Samples were brought to the hospital the day after the collection, they were immediately frozen at − 80 °C until nucleotide extraction. FIT and stool samples were stored at -80˚C for approximately 3–5 years prior to RNA extraction. Colonoscopy was recommended because the patients had abnormal or positive FIT results (i.e., there was blood in the stool at the time of the test). AAs﻿﻿ were defined based on the presence of high-grade dysplasia, villous component, or lesion size of > 1 cm, as defined by Zarchy and Ershoff [11].

### Small RNA extraction and library preparation

FIT stool samples were obtained from buffer leftovers contained in the original collection device (approximately 1ml). NORCCAP feces was thawed and homogenized in a buffer (Omnigene-GUT, DNAgenotek). For BCSN FIT and NORCCAP stool samples, RNA was extracted from 200 µl buffer leftovers and buffer mix, respectively. RNA was purified using phenol-chloroform phase separation and miRNeasy Mini Kit (cat. no. 217,004, Qiagen).

For the MITOS cohort, total RNA from stool and FIT leftover samples was extracted using 200 µl input material and the Stool Total RNA Purification Kit (Norgen Biotek Corp.) as previously described [7, 8].

sncRNA transcripts were converted into barcoded cDNA libraries with the NEBNext Multiplex Small RNA Library Prep Set for Illumina following the NEBNext Multiplex Small RNA Library Prep (Protocol E7330, New England BioLabs Inc., USA) [7, 12].

The size selection of purified RNA fragments for the MITOS cohort was performed as described in [7]. For BCSN and NORCCAP samples the size selection was performed with a cut size optimized to cover RNA molecules from 17 to 47 nt in length. Small RNA libraries were indexed and sequenced on Illumina platforms.

One miRNA, miR-1246, was validated in MITOS-FIT samples by quantitative Real-time PCR (qRT-PCR)(details in Supplementary Methods).

### Bioinformatics and statistical analysis

Reads were quality filtered and adapters removed with fastp in default settings. Mapping of reads on miRBase v22.1 miRNA sequences was performed using smrnaseq (https://nf-co.re/smrnaseq) with *skip_mirdeep* option. miRNA-unmapped reads were aligned against the human hg38 genome using Bowtie2 with *–very-sensitive-local* option. The human-unmapped reads were mapped against microbial genomes using Kraken 2 (v2.1.2) as described in [7].

miRNA read count normalization and Differential Expression (DE) analyses were performed with DESeq2. Differential abundance analysis of reads mapped to microbes was performed with SIAMCAT with default settings. For more details, see Supplementary Methods. Concordance between sampling methods was evaluated by comparing the mean abundance of features (miRNAs or microbial species) across datasets. Correlation analyses were performed using the Spearman correlation method.

## Results and discussion

### Cohort and alignment statistics

**Fig. 1:**
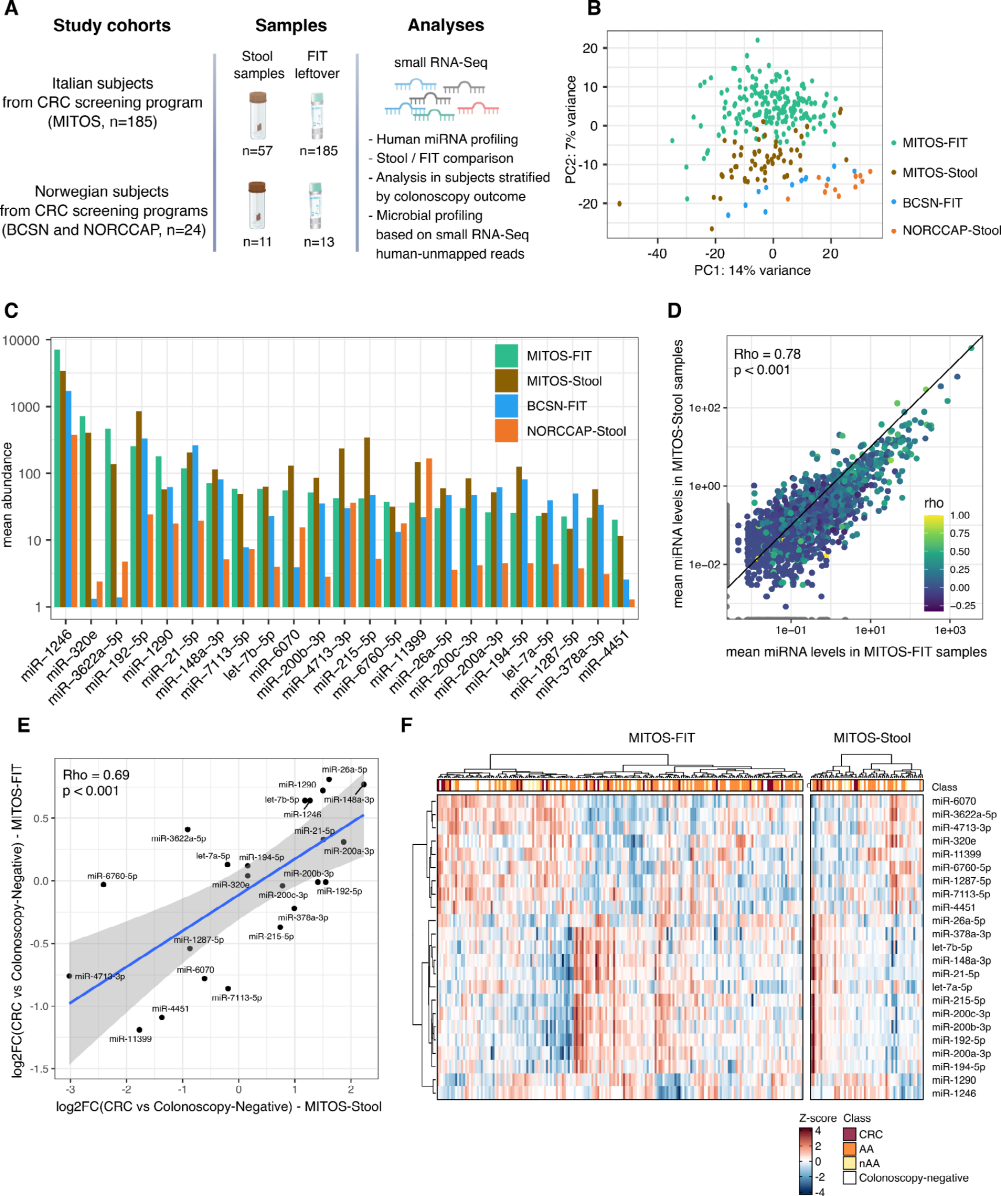
** A.** Graphical representation of the study. **B**. Principal component analysis (PCA) of samples based on mature miRNA read counts. **C.** Bar plot reporting, for each cohort and sample type, the average levels of the miRNAs associated with the highest levels in MITOS-FIT leftover samples. **D.** Mean normalised abundance of miRNAs in paired MITOS-FIT (x-axis) and MITOS-Stool (y-axis) samples. Each point represents a miRNA coloured based on the Spearman correlation coefficient for comparison of paired samples. Only miRNAs detected in at least 15% of each sample type were included in this analysis. **E.** Scatterplot showing the log2FC of expression computed considering MITOS-stool (x-axis) and MITOS-FIT leftover (y-axis) miRNA levels in samples from CRC patients with respect to those from colonoscopy-negative subjects. **F.** Heatmap of the Z-score normalised miRNA levels in MITOS-stool and MITOS-FIT leftover samples. AA, advanced adenoma; nAA, non-advanced adenoma; CRC, colorectal cancer

Fecal samples collected with different sampling, storage, and processing procedures were analysed to identify stably detectable miRNAs with a potential to be used as CRC biomarkers (Fig. 1A). From the MITOS cohort, two sample types were collected for each individual: stool samples collected in RNA-stabilising buffer, and leftover buffer derived from CRC-screening FIT samples. Two sets of anonymized Norwegian samples were also assessed: archived stool samples stored without stabilising buffer from the NORCCAP study, and leftover buffer from Norwegian CRC-screening FIT samples from the BCSN trial.

As reported in **Supplementary Table 1 A** and **B**, a mean of 0.12% (range: 0.003–0.62%) and 0.15% (range:0.03–0.61%) of small RNA sequencing reads were assigned to miRNAs, respectively, in stool samples and in FIT leftover buffers. Considering 10 as the minimum number of normalised reads to define a miRNA as detected, on average 63 (range: 32–235) and 41 (range: 16–191) miRNAs were detected in stool and FIT leftover samples, respectively (**Supplementary Table 1 A-B**). Still, when accounting for differences in sequencing depth by rarefaction, no differences in the number of miRNAs detected were found between sampling groups (p > 0.1). The stool miRNAs detected in the MITOS cohort samples included most of the annotations observed in previous analyses performed on the same samples analysed using a different pipeline [7, 8].

For two FIT leftover samples, small RNA sequencing was performed on libraries generated from two different amounts of starting material (250 and 400 µl). Comparing the rate of miRNA-mapped reads, similar rates were observed (0.4–0.9% of mapped reads) with an average of 33 miRNAs (range: 31–34) consistently detected in each experiment. No significant differences were observed among miRNA levels measured in such experiments and, as expected, the levels of detected molecules were significantly correlated (rho = 0.43–0.60, p < 0.001) (**Supplementary Fig. 1A** and **Supplementary Table 1 C**). The intra-individual correlation between miRNA levels was higher than among different individuals (**Supplementary Fig. 1B**). Overall, differences were observed in miRNA profiles related both to sampling procedure and to participant population (see PCA analysis in Fig. 1B).

Since the 57 stool samples from the MITOS cohort were collected from the same subjects donating FIT leftover samples, a paired comparative analysis was performed between the miRNA levels measured in the two biospecimens. The analysis was focused on the levels of 23 miRNAs that were consistently identified across both stool and FIT samples (exceeding a median of 10 normalised reads; Fig. 1C and **Supplementary Table 1D**). Most of these miRNAs (n = 21) were previously detected by us in an analysis of stool miRNome and nine of them (miR-1246, -21-5p, -26a-5p, -148a-3p, let-7b-5p, -200b-3p, -194-5p, -1290) overlapped with a set of 25 stool miRNAs whose levels were consistently dysregulated in sporadic CRC patients from different European populations [8]. Furthermore, these miRNAs correspond well with the most abundant miRNAs in circulation, including miR-1246, -320, -21-5p, -1290, -148a-3p being among the most abundant miRNAs in serum samples [13] and plasma extracellular vesicles (EVs) samples [8]. In the latter study, miR-1246 was also significantly more abundant in plasma EVs of CRC cases than those from colonoscopy-negative controls [8]. As reported in **Supplementary Table 1D**, levels of 57% of these miRNAs were positively correlated (average rho = 0.36, p < 0.05) between the two biospecimens. Among them, miR-4713-3p, miR-1246, and miR-192-5p were characterised by the highest correlation. All the 23 miRNAs were also detected in the Norwegian cohorts (Fig. 1C), despite the different sampling, preservation, and RNA extraction procedures. Still, the abundances observed in the NORCCAP samples were low compared to the other cohorts, and likely result from long storage times and lack of preservation buffer. Storage effects have been identified previously when assessing the microbiome in this cohort [4].

There was a positive correlation between paired FIT and stool samples for the mean normalised abundance of miRNAs (rho = 0.78, p < 0.001; Fig. 1D), which was also found for the unpaired Norwegian samples (rho = 0.52, p < 0.001) (**Supplementary Fig. 1C**). These data confirm that putative miRNAs can be consistently detected in both archived and newly-collected samples from different populations.

### miRNA differential expression in MITOS-stool and MITOS-FIT samples

DE analysis was performed between stool and FIT leftover miRNA levels detected in MITOS subjects with CRC or AA (considered separately or together, CRC + AA) with respect to colonoscopy-negative subjects. Considering the 23 miRNAs detected in both biofluids, 12 were associated with significantly different levels in stool samples from CRC or AA patients (adj. p < 0.05; **Supplementary Table 1E**). Comparing separately AA and CRC patients with colonoscopy-negative subjects, four and eight DE miRNAs were observed, respectively. Interestingly, let-7b-5p was DE in both comparisons. In addition, four miRNAs were significantly more (let-7b-5p and miR-148a-3p) and less (miR-4451 and miR-11399) abundant in FIT leftover samples of CRC patients with respect to colonoscopy-negative subjects (**Supplementary Table 1F)**. The levels of the 23 miRNAs were characterized by a coherent difference in both MITOS-stool and MITOS-FIT samples (rho = 0.69, p < 0.001) (Fig. 1E). Clustering analysis of the miRNA levels in FIT leftover and stool samples of the MITOS cohort showed two main miRNA clusters and a partial separation between colonoscopy-negative subjects and AA or CRC patients (Fig. 1F).

To technically validate the presence of miRNAs in FIT buffer leftover samples, the levels of miR-1246 were evaluated by qRT-PCR in samples from 38 subjects of the MITOS cohort (5 CRC, 19 AA, 6 nAA patients and 8 colonoscopy-negative controls). The analysis confirmed the presence of this miRNA in all the analyzed samples (Ct < 33, **Supplementary Table 1G**). In addition, the miR-1246 FIT leftover levels measured by qRT-PCR were significantly related with those detected by sRNA-Seq performed on the same sample (rho = 0.69, p < 0.001, **Supplementary Fig. 1D**). These results are consistent with our previous qRT-PCR validation of miRNA detection in stool samples [8].

Functional analysis of DE miRNA target genes showed the prevalence of terms related to cell cycle regulation and DNA-damage response for the targets of miRNAs with increased levels in CRC patients (**Supplementary Table 1 H, I**). Conversely, targets of miRNAs decreasing in patient samples were enriched in processes related to apoptosis, unfolded protein stress response, and immune response (**Supplementary Table 1 H I**).

### Microbial profiling based on small RNAs

After the identification of human miRNAs, the remaining reads from sequencing were aligned against the human genome and the subsequent unmapped reads were investigated for their microbial sncRNA content. This approach classified reads in the range of 36–40 nt, of which 38.5% and 37.4% were classified in the MITOS stool and FIT samples, respectively.

**Fig. 2:**
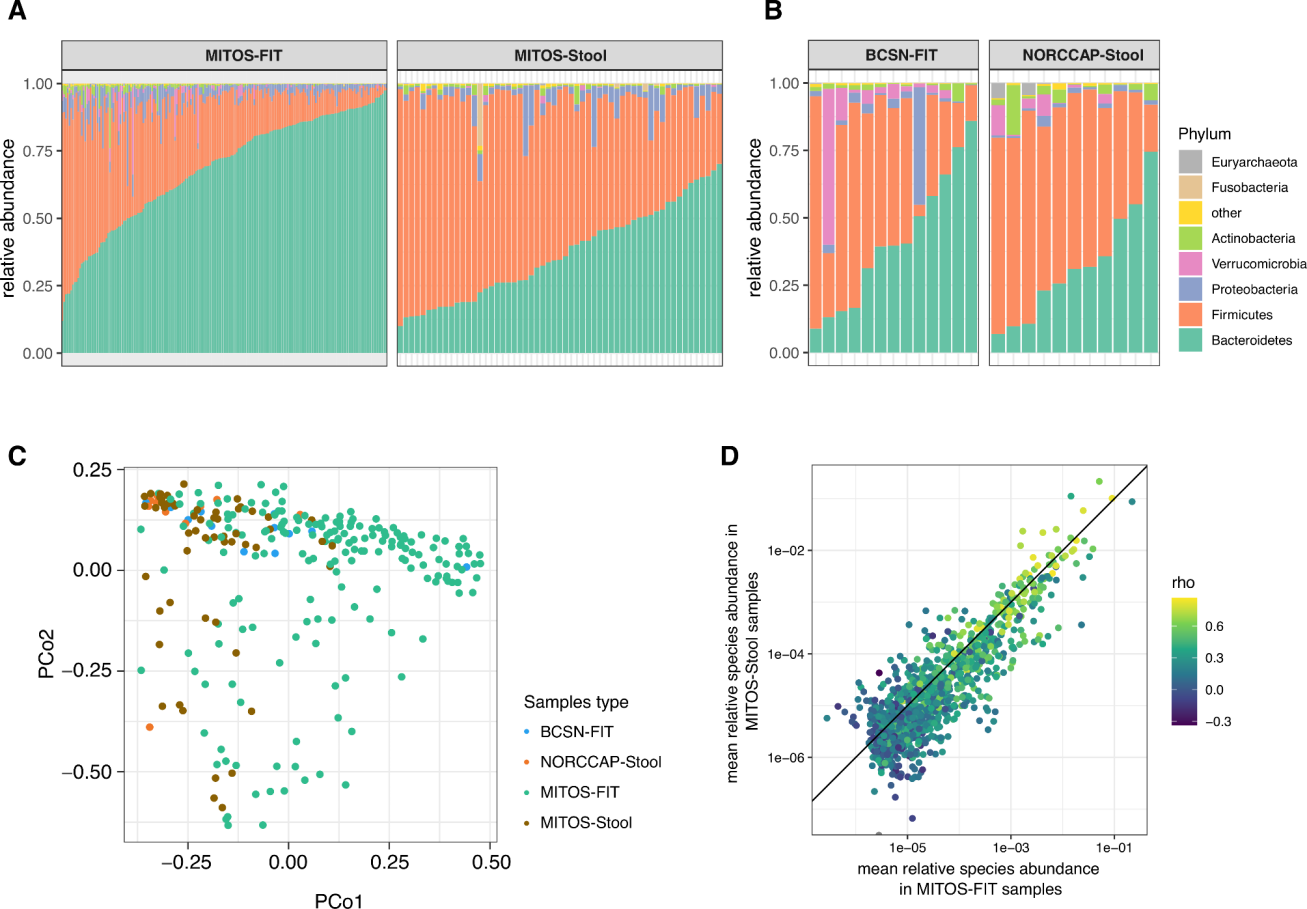
** A-B.** Classification of reads according to microbial taxa. Relative abundance of phyla in FIT leftover and stool samples from MITOS (**A**), BCSN,  and NORCCAP (**B**) cohorts. **C.** Principal coordinate (PCo) plot of FIT and stool samples (including unpaired samples). **D**. Mean relative abundance of species in stool samples (y-axis) and FIT samples (x-axis), coloured according to the Spearman’s rho of the correlation between the relative abundances in paired FIT and stool samples. The solid diagonal line represents equal abundance in either biospecimen type

Given the previous evidence on the concordance between microbial abundances estimated by small RNA sequencing and metagenomic data [7], the human-unmapped sRNA-Seq reads were used to infer the microbial abundance in our data. Overall, FIT samples displayed a higher abundance of taxa belonging to the *Bacteroidetes* phylum, whereas stool samples were dominated by *Firmicutes* (Fig. 2A-B), with the composition of stool and FIT samples differing significantly (PERMANOVA p < 0.05; Fig. 2C). This could indicate a differential sensitivity of bacteria to the buffer components in the FIT and Norgen buffers, where the former has a relatively high concentration of the potent antimicrobial compound sodium azide [14]. Still, at the species level, there was concordance between FIT and stool samples (Fig. 2D). Within the MITOS study subjects, we assessed differences in microbial taxa between colonoscopy-negative and either CRC, AA, or CRC/AA (**Supplementary Table 1 J)**, and although they were not statistically significant, the direction and magnitude of differences in taxa abundance between CRC cases and colonoscopy-negative subjects were consistent in stool and FIT samples (rho = 0.53, p = 0.002; **Supplementary Fig. 2**).

## Conclusions

Taken together, our results show that by using small RNA sequencing we can profile both stool miRNAs and microbial taxa in the left-over FIT buffer used in CRC screening. The consistent levels of miRNAs between sampling methods suggest that FIT may be used for miRNA biomarker research in large scale screening settings. This feasibility study also confirms that the alterations in gut miRNA levels in CRC patients observed in FIT samples may be used to detect miRNAs in FIT as biomarkers to improve screening performance.

### Electronic supplementary material

Below is the link to the electronic supplementary material.

Supplementary Material 1

Supplementary Material 2

Supplementary Material 3

## Data Availability

The datasets used and/or analysed during the current study are available from the corresponding author on reasonable request.
