# Supplementary material for: Profiling small RNAs in fecal immunochemical tests: is it possible?
Source: Mol Cancer. 2023 Oct 3;22:161. doi: 10.1186/s12943-023-01869-w (PMC10546694; doi:10.1186/s12943-023-01869-w)
Supplement: Supplementary file 3 — Supplementary Material 3 [file 12943_2023_1869_MOESM3_ESM.pdf]

Profiling small RNAs in fecal immunochemical tests: is it possible?

Einar Birkeland*, Giulio Ferrero*, Barbara Pardini, Sinan U. Umu, Sonia Tarallo, Sara Bulfamante, Geir Hoff, Carlo Senore, Trine B Rounge*, Alessio Naccarati*

*Equal contribution

Supplementary Figures

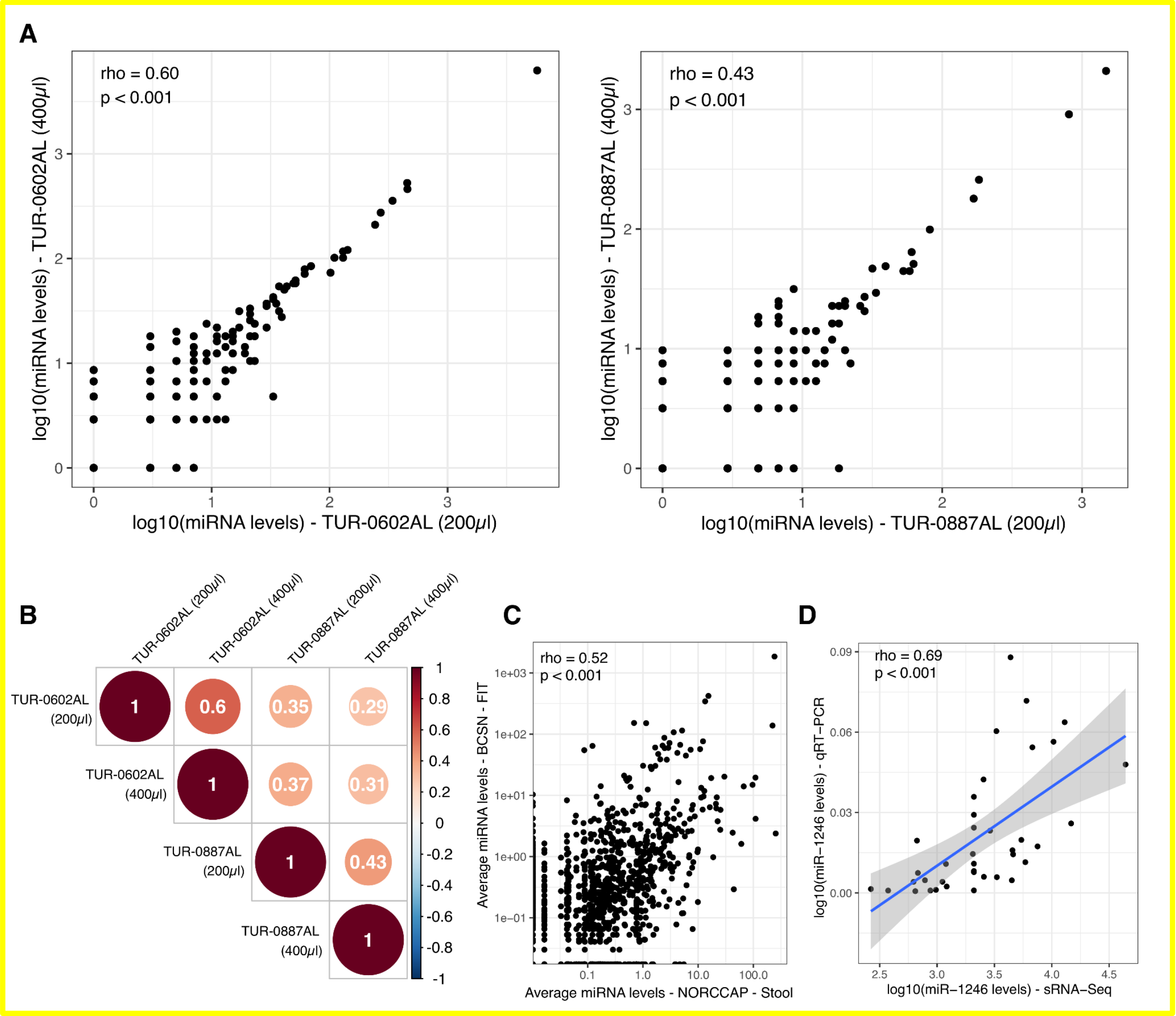

Supplementary Figure 1. A. Scatter plot showing the log10 normalized miRNA levels measured by the small RNA-Seq performed on samples with different starting volumes for small RNA extraction. **B.** Correlation heat map showing the Spearman correlation coefficient computed between miRNA levels measured in FIT leftover samples from the same subjects with different starting volumes; dot size represents the spearman correlation value, the color indicates the verse of correlation. **C.** Scatter plot reporting the average miRNA levels detected in stool and FIT leftover samples from the BCSN and NORCCAP cohorts, respectively. **D.** Scatterplot of the miR-1246 levels measured by sRNA-Seq and qRT-PCR on FIT buffer leftover samples from the same subject.

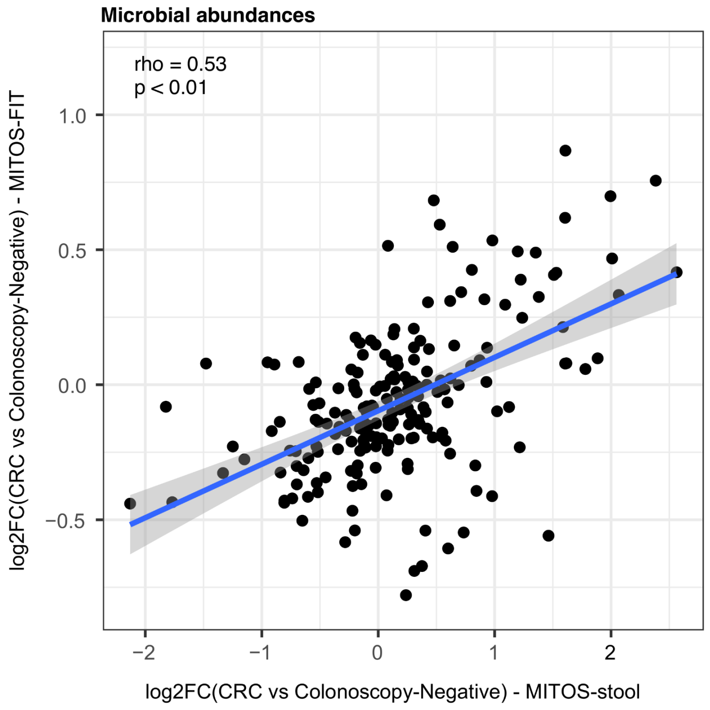

Supplementary Figure 2. Scatterplot showing the log2-transformed fold change (log2FC) for differences in microbial abundance between CRC cases and colonoscopy negative subjects in the MITOS study when measured using stool samples (x-axis) and FIT leftover buffer (y-axis).

Supplementary Methods

Sample collection

FIT stool samples were obtained from buffer leftovers contained in the original collection device (approx. 1ml). For the Italian and BCSN FIT cohorts, buffer leftovers were collected a few hours from Hb measurements and immediately divided respectively in 250µl and 200µl aliquots and preserved at -80°C and -40°C until use. Aliquots were thawed the day of the RNA/DNA extraction. NORCCAP feces were thawed and homogenized in a buffer (Omnigene-GUT, DNAgenotek).

Extraction of RNA from FIT and stool samples.

For BCSN FIT and NORCCAP stool samples, RNA was extracted from 200µl buffer leftovers and buffer mix, respectively. RNA was purified using phenol-chloroform phase separation and miRNeasy Mini Kit (cat. no. 217004, QIAgen) on a Qiacube. Glycogen (Cat. no AM9510, Invitrogen) was used as a carrier during the RNA extraction step. Final elution volume was 30µl and concentration was quantified by Qubit fluorometer (Invitrogen).
For the MITOS cohort, total RNA from stool and FIT leftover samples was extracted from 200ul input material using the Stool Total RNA Purification Kit (Norgen Biotek Corp.) as previously described [1, 2]. For two samples from MITOS cohort, RNA extraction was performed also from 400µl aliquot of FIT leftover with the aim to evaluate if RNA yield could be increased and to test if there were any differences in the small RNA-seq data according to the different starting volume. The RNA concentration was quantified by Qubit microRNA Assay Kit

(Invitrogen) with Qubit fluorometer (Invitrogen), according to the MIQE guidelines (http://miqe.gene-quantification.info/).

Library preparation for small RNA-sequencing (small RNA-seq)

For the MITOS cohort, the size selection and purification of the amplified cDNA constructs were performed following the instructions described in the Illumina TruSeq small RNA library prep protocol and in [1, 2]. The 140-nt and 150-nt bands correspond to adapter-ligated constructs derived from RNA fragments of 21 to 30 nt. Pools of libraries (30-plex) were sequenced on an Illumina NextSeq500 (75 cycles, single end; Illumina Inc).

For BCSN and NORCCAP samples, the size selection was performed using a 3% Agarose Gel Cassette (Cat. No CSD3010) on a Pippin Prep (Sage Science) with a cut size optimized to cover RNA molecules from 17 to 47 nt in length. Sequencing libraries were indexed and 12 samples were sequenced per lane of a HiSeq 2500 (Illumina).

Quantitative Real-Time Polymerase Chain Reaction (qRT-PCR)

To technically-validate the presence of miRNAs in FIT buffer leftover samples assessed by small RNA sequencing, miR-1246 levels were measured in MITOS-FIT samples (n=38) using the miRCURY LNA SYBR Green PCR kit (Qiagen), according to the manufacturer's instructions for plasma/serum. Reverse transcription (RT) was performed using the miRCURY LNA RT kit (Qiagen) according to the manufacturer's instructions with the addition of 1 spike-in (UniSp6) to the RT reaction. For qPCR, complement cDNA was diluted 1:30; 3 µL of 1:30 water-diluted cDNA products were mixed at 7 µL of miRCURY SYBR Green Mastermix containing 1 µL of specific miRNA probe (Qiagen). All cDNA products were prepared in triplicate PCR reactions following the manufacturer's instructions. For quality control purposes, one RNA sample was measured twice, and a sample containing nuclease-free water and carrier RNA was profiled as the negative control. All the reactions were run on the ABI Prism 7900 Sequence Detection System (Applied Biosystems). A melt curve analysis was performed for the amplification specificity of each individual target per sample.

The expression levels of miR-1246 were normalized to UniSp6 using the 2-ΔCt and 2-ΔΔCt formulas. Ct values were normalized by subtracting the Ct value of the selected endogenous controls from the miRNA of interest. Differential miRNA expression was determined by the Wilcoxon Rank-Sum test. P-values< 0.05 were considered as statistically significant.

Bioinformatics and statistical analysis

Reads mapping to miRNAs were normalized using the DESeq2 [3] function "estimate size factors". For PCA analysis, vst transformation of miRNA counts was used. For rarefaction, the estimateD function of the R package iNEXT [4] was used to estimate miRNA richness. miRNA DESeq2 Differential Expression (DE) analyses were performed, considering only miRNAs with a median of 10 or more normalized reads in both FIT and stool samples.

Microbial alpha and beta diversity were measured using the inverse Simpson index, and Bray Curtis dissimilarity metric, respectively, both implemented using the R package vegan [5, 6]. SIAMCAT analysis was performed after removing features with median relative abundance > 0.001 in both MITOS-FIT and MITOS-stool.

References

1. Tarallo S, Ferrero G, Gallo G, Francavilla A, Clerico G, Realis Luc A, Manghi P, Thomas AM, Vineis P, Segata N, et al: **Altered Fecal Small RNA Profiles in Colorectal Cancer Reflect Gut Microbiome Composition in Stool Samples.** *mSystems* 2019, **4**.
2. Pardini B, Ferrero G, Tarallo S, Gallo G, Francavilla A, Licheri N, Trompetto M, Clerico G, Senore C, Peyre S, et al: **A Fecal MicroRNA Signature by Small RNA Sequencing Accurately Distinguishes Colorectal Cancers: Results From a Multicenter Study.** *Gastroenterology* 2023, **165:**582-599 e588.
3. Love MI, Huber W, Anders S: **Moderated estimation of fold change and dispersion for RNA-seq data with DESeq2.** *Genome Biol* 2014, **15:**550.
4. Hsieh TC, Ma KH, Chao A: **iNEXT: an R package for rarefaction and extrapolation of species diversity (Hill numbers).** *Methods in Ecology and Evolution* 2016, **7:**1451-1456.
5. Wirbel J, Pyl PT, Kartal E, Zych K, Kashani A, Milanese A, Fleck JS, Voigt AY, Palleja A, Ponnudurai R, et al: **Meta-analysis of fecal metagenomes reveals global microbial signatures that are specific for colorectal cancer.** *Nat Med* 2019, **25:**679-689.
6. Thomas AM, Manghi P, Asnicar F, Pasolli E, Armanini F, Zolfo M, Beghini F, Manara S, Karcher N, Pozzi C, et al: **Metagenomic analysis of colorectal cancer datasets identifies cross-cohort microbial diagnostic signatures and a link with choline degradation.** *Nat Med* 2019, **25:**667-678.
